# Results of the phase IIa study to evaluate the efficacy and safety of rezivertinib (BPI-7711) for the first-line treatment of locally advanced or metastatic/recurrent NSCLC patients with *EGFR* mutation from a phase I/IIa study

**DOI:** 10.1186/s12916-022-02692-8

**Published:** 2023-01-08

**Authors:** Yuankai Shi, Jianying Zhou, Yanqiu Zhao, Bo Zhu, Liangming Zhang, Xingya Li, Jian Fang, Jianhua Shi, Zhixiang Zhuang, Sheng Yang, Donglin Wang, Huiqing Yu, Longzhen Zhang, Rongsheng Zheng, Michael Greco, Tingting Wang

**Affiliations:** 1 Department of Medical Oncology, National Cancer Center/National Clinical Research Center for Cancer/Cancer Hospital, Chinese Academy of Medical Sciences & Peking Union Medical College, Beijing Key Laboratory of Clinical Study on Anticancer Molecular Targeted Drugs, No. 17 Panjiayuan Nanli, Chaoyang District, Beijing, 100021 China; 2 Department of Respiratory Medicine, The First Affiliated Hospital, Zhejiang University, School of Medicine, Hangzhou, China; 3 Department of Respiratory Medicine, The Affiliated Cancer Hospital of Zhengzhou University, Zhengzhou, China; 4 Department of Oncology, Institute of Cancer, Xinqiao Hospital, Third Military Medical University, Chongqing, China; 5 Department of Medical Oncology, Yantai Yuhuangding Hospital, Yantai, China; 6 Department of Oncology, The First Affiliated Hospital of Zhengzhou University, Zhengzhou, China; 7 Department of Thoracic Oncology, Beijing Cancer Hospital, Beijing, China; 8 Department of Medical Oncology, Linyi Cancer Hospital, Linyi, China; 9 Department of Oncology, The Second Affiliated Hospital of Soochow University, Suzhou, China; 10 Department of Medical Oncology, Chongqing University Cancer Hospital, Chongqing, China; 11 Department of Palliative Care, Department of Geriatric Oncology, Chongqing University Cancer Hospital, Chongqing, China; 12 Department of Radiotherapy, The Affiliated Hospital of Xuzhou Medical University, Xuzhou, China; 13 Department of Medical Oncology, The First Affiliated Hospital of Bengbu Medical College, Bengbu, China; 14 Department of Drug Discovery, Beta Pharma Inc., Princeton, NJ USA; 15 Department of Clinical Development, Beta Pharma (Shanghai) Co., Ltd, Shanghai, China

**Keywords:** Rezivertinib, BPI-7711, NSCLC, *EGFR* mutation, Third-generation EGFR TKI

## Abstract

**Background:**

Rezivertinib (BPI-7711) is a novel third-generation epidermal growth factor receptor (EGFR) tyrosine kinase inhibitor (TKI). This phase IIa study was part of a phase I/IIa study (NCT03386955), aimed to evaluate the efficacy and safety of rezivertinib as the first-line treatment for patients with locally advanced or metastatic/recurrent *EGFR* mutated non-small cell lung cancer (NSCLC).

**Methods:**

Patients received the first-line treatment of 180 mg rezivertinib orally once daily until disease progression, unacceptable toxicity, or withdrawal of consent. The primary endpoint was the objective response rate (ORR) assessed by blinded independent central review (BICR). Secondary endpoints included disease control rate (DCR), duration of response (DoR), progression-free survival (PFS), overall survival (OS), and safety.

**Results:**

From Jun 12, 2019, to Oct 17, 2019, 43 patients were enrolled. At the data cutoff date on Dec 23, 2021, the ORR by BICR was 83.7% (95% CI: 69.3–93.2%). The median DoR was 19.3 (95% CI: 15.8–25.0) months. The median PFS by BICR was 20.7 (95% CI: 13.8–24.8) months and 22.0 (95% CI: 16.8–26.3) months by investigators. Data on OS was immature. Totally, 40 (93.0%) patients had at least one treatment-related adverse event while 4 (9.3%) of them were grade ≥ 3.

**Conclusions:**

Rezivertinib (BPI-7711) showed promising efficacy and a favorable safety profile for the treatment among the locally advanced or metastatic/recurrent NSCLC patients with *EGFR* mutation in the first-line setting.

**Trial registration:**

ClinicalTrials.gov, NCT03386955.

**Supplementary Information:**

The online version contains supplementary material available at 10.1186/s12916-022-02692-8.

## Background

Lung cancer is the second most commonly diagnosed cancer worldwide but leading the cause of cancer death [1]. Non-small cell lung cancer (NSCLC) comprises about 80 to 85% of all lung cancers, with the majority of patients presenting with locally advanced or metastatic disease [2, 3]. Epidermal growth factor receptor (*EGFR)* mutations are found in up to 10% of Caucasians, and more than 40% of East Asian adenocarcinoma patients [4–6]. According to the latest National Comprehensive Cancer Network (Version 3.0, 2022) and the Chinese guideline, EGFR tyrosine kinase inhibitors (TKIs) are the current first-line treatments for NSCLC with *EGFR* mutation [7, 8], including the first-generation EGFR TKIs with gefitinib, erlotinib, and icotinib; the second-generation EGFR TKIs with afatinib and dacomitinib; and the third-generation EGFR TKIs with osimertinib, almonertinib, and furmonertinib. As reported, after around 1-year treatment of the first- or second-generation EGFR TKIs, most patients acquired drug resistance [9, 10]. *EGFR* T790M mutation occurs in more than 50% of the patients showing acquired drug resistance [11]. Osimertinib, a third-generation EGFR TKI, was approved by the US Food and Drug Administration (FDA) on November 13, 2015, for advanced *EGFR* T790M mutated NSCLC [12]. Almonertinib and furmonertinib were first approved by the National Medical Products Administration (NMPA) in the People’s Republic of China on Mar 17, 2020, and Mar 3, 2021, respectively [13–16]. Meanwhile, the clinical development for novel third-generation EGFR TKIs is ongoing widely due to the high proportion of *EGFR*-mutant patients and diversified features of different third-generation EGFR TKIs [12].

Rezivertinib (BPI-7711) is a novel third-generation EGFR TKI jointly developed by Beta Pharma (Shanghai) Co., Ltd., Shanghai, People’s Republic of China, and Beta Pharma Inc., Princeton, NJ, USA, which can selectively target specific mutated *EGFR* and form irreversible covalent binding at the active binding site, showing the highly selective inhibitory effect on *EGFR* mutation such as exon 19 deletion, L858R point mutation, T790M mutation, and the weak inhibitory effect on *EGFR* wild-type. In a previous phase I study, rezivertinib resulted in an objective response rate (ORR) of 59.3%, a disease control rate (DCR) of 91.3%, and a median progression-free survival (PFS) of 9.7 months for advanced NSCLC patients with *EGFR* T790M mutation, and the recommended phase II dose (RP2D) was identified as 180 mg once daily [17]. The phase IIb study results further revealed the promising efficacy with a manageable safety profile of rezivertinib for patients with locally advanced or metastatic/recurrent *EGFR* T790M-mutated NSCLC [18]. This phase IIa study was part of the phase I/IIa study. In this study, we evaluate the efficacy and safety of rezivertinib in the first-line treatment of locally advanced or metastatic/recurrent NSCLC patients with *EGFR* mutation.

## Methods

### Study design and patients

This was a multicenter, single-arm, open-label, phase IIa study, which was part of the phase I/IIa study (NCT03386955), conducted across 20 hospitals in the People’s Republic of China. Key inclusion criteria included patients aged 18–75 years; with a histologically or cytologically confirmed locally advanced or metastatic NSCLC harboring *EGFR*-sensitive mutations, including exon 19 deletion, L858R, G719X, and L861Q which were detected through tissue or/and plasma biopsies by central laboratory testing using the Cobas® *EGFR* Mutation Test, Version 2, Roche Diagnostics, South Branchburg, NJ, USA; who have at least one measurable lesion; with an Eastern Cooperative Oncology Group (ECOG) performance status (PS) score of 0 to 1; who have no disease deterioration over the previous 2 weeks; and with at least a 12-week life expectancy who were not suitable for operation or radiotherapy. Patients with previous neoadjuvant or adjuvant therapies including chemotherapy, radiotherapy, and investigational drug were acceptable, except EGFR TKIs. Adequate organ function was required as defined by platelet (PLT) count ≥ 100 × 10^9^/L, absolute neutrophil count (ANC) ≥ 1.5 × 10^9^/L, hemoglobin ≥ 90 g/L, total bilirubin ≤ 1.5 × the upper limit of normal (ULN), alanine aminotransferase (ALT) and aspartate aminotransferase (AST) ≤ 3 × ULN (total bilirubin ≤ 3 × ULN, ALT ≤ 5 × ULN, and AST ≤ 5 × ULN were allowed if liver metastases existed), serum creatinine ≤ 1.5 × ULN, or creatinine clearance ≥ 50 mL/min according to the Cockcroft-Gault equation, QT interval corrected for heart rate using Fridericia’s formula (QTcF) prolongation ≤ 470 ms at rest, international normalized ratio (INR) and activated partial thromboplastin time (APTT) ≤ 1.5 × ULN without taking an anticoagulant. Before the first dose of rezivertinib, all prior treatment-related adverse events (TRAEs) had to be at grade ≤ 1 except for hair loss and peripheral nerve toxic reaction. The ability to swallow capsules was required.

Key exclusion criteria included patients who received previous anticancer treatment for advanced NSCLC including EGFR TKIs, cytotoxic chemotherapy, and investigational agent; any clinically significant electrocardiogram (ECG) abnormality (such as QTcF prolongation > 470 ms at rest, complete left bundle branch block); any factor that increased the risk of QTcF prolongation (such as New York Heart Association II-IV, hypokalemia, long QT syndrome); any condition that possibly affected drug absorption such as severe or uncontrolled inflammatory gastrointestinal disease, abdominal colostomy, gastrointestinal perforation within 6 months, extensive bowel resection, or tube feeding patients; medical history of interstitial lung disease (ILD), radiation pneumonitis that required steroid treatment, and acute or progressive lung disease that could lead to ILD; active infection disease such as hepatitis B, hepatitis C, and human immunodeficiency virus, inactive hepatitis B was acceptable; major surgery within 4 weeks, minor operation within 2 weeks; radiotherapy with a wide field within 4 weeks, or radiotherapy within a limited field within 1 week before the first dose of rezivertinib; patients with any other concomitant cancer or recurrent cancer within 5 years, except radical operation of carcinoma in situ of cervix, non-melanoma skin cancer, noninvasive superficial bladder cancer, or radical operation of carcinoma in situ with no recurrence within 3 years; patients with spinal cord compression or meningeal metastases, symptomatic brain metastases, except asymptomatic brain metastases not requiring steroids and/or local therapy before this study, asymptomatic brain metastases after local therapy such as radiotherapy and steroids and/or antiepileptic therapy at least 7 days before the first dose of rezivertinib.

Informed consent was obtained from every patient before enrollment. The study was done in accordance with the Declaration of Helsinki and approved by the institutional review board or independent ethics committee associated with each participating hospital.

### Procedures

Eligible patients received rezivertinib 180 mg orally once daily until disease progression, unacceptable toxicity, or withdrawal of consent. Treatment beyond progression was permitted if clinical benefits could be obtained in the judgment of the investigators.

Dose adjustment was allowed according to such principles. If a patient had a grade ≥ 3 TRAE, the administration of rezivertinib should be suspended, and supportive care should be given accordingly. After the grade ≥ 3 TRAE was relieved or recovered to grade ≤ 1 within 2 weeks after dose interruption, the investigators would restart the treatment at the initial dose (180 mg) or a lower dose (120 mg → 60 mg) according to the patient’s condition, and close medical monitoring was necessary.

Efficacy was assessed by blinded independent central review (BICR) and by investigators with enhanced computed tomography scans for the chest and abdomen or magnetic resonance imaging scans for the brain at baseline and every 2 treatment cycles (6 weeks) from the first dose of rezivertinib. In the period between the time when the informed consent was signed and 30 days after the last dose of rezivertinib, adverse events (AEs) were monitored continuously. During the treatment period, physical examinations, vital signs, ECOG PS scores, hematology, serum chemistry, urinalysis, 12-lead ECGs, and echocardiography were documented and assessed at protocol-specified time points.

### Endpoints and assessments

The primary endpoint was ORR in full analysis set (FAS) evaluated by BICR per Response Evaluation Criteria in Solid Tumors (RECIST) version 1.1 [19]. The efficacy for patients with central nervous system (CNS) metastases was measured by BICR according to the Response Assessment in Neuro-Oncology Brain Metastases (RANO-BM) criteria [20]. The secondary endpoints included DCR, duration of response (DoR), and PFS assessed by both BICR and investigators; overall survival (OS); safety assessed by investigators. Safety referred to treatment-emergent adverse events (TEAEs) and TRAEs which were assessed in the safety set (SS) according to the National Cancer Institute Common Terminology Criteria for Adverse Events (CTCAE) version 4.03.

FAS referred to all patients enrolled who received at least one dose of rezivertinib. SS included all patients who received at least one dose of rezivertinib and had safety data available. ORR was defined as the proportion of patients with confirmed complete response (CR) or partial response (PR). DCR was defined as the proportion of patients who had confirmed CR, PR, or stable disease (SD). DoR was defined as the time from the first CR or PR to disease progression or death which applied only to patients with confirmed CR or PR. PFS was defined as the time from the first dose of rezivertinib until the earliest date of documented disease progression or death due to any cause. OS was defined as the time from the date of the first dose of rezivertinib until death due to any cause. CNS-ORR was defined as the proportion of confirmed CR or PR in brain metastatic lesions, as evaluated by BICR according to the RANO-BM criteria. CNS-DCR was defined as the proportion of confirmed CR, PR, or SD in brain metastatic lesions evaluated by BICR according to the RANO-BM criteria.

### Statistical analysis

The 95% confidence interval (CI) for ORR and DCR was determined by the Clopper-Pearson method. The 95% CI for median values of PFS, DoR, and OS was calculated by the Kaplan–Meier method. All statistical analyses were performed using SAS® Version 9.3 or higher.

## Results

### Patients

**Fig. 1:**
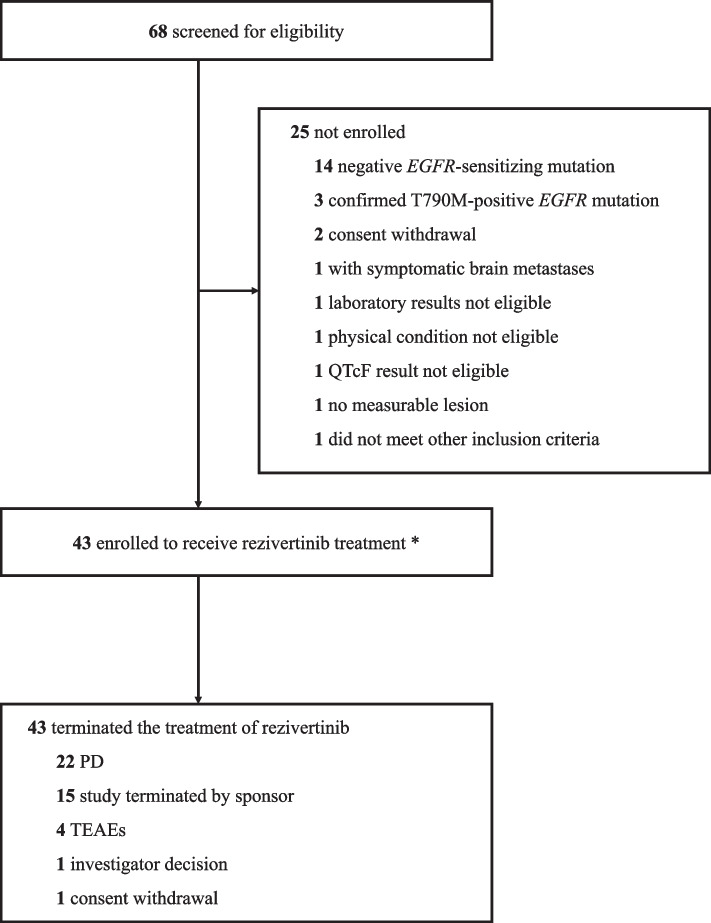
Patient disposition. *Two patients accompanied with T790M mutation were mistakenly enrolled, one patient was with T790M positive and exon 19 deletion mutation and the other was with T790M positive and L858R mutation. Abbreviations: *EGFR*, epidermal growth factor receptor; QTcF, QT interval corrected for heart rate using Fridericia’s formula; PD, progressive disease; TEAEs, treatment-emergent adverse events

From Jun 12, 2019, to Oct 17, 2019, 68 patients were screened. Finally, a total of 43 eligible patients were enrolled, and all patients received rezivertinib treatment (Fig. 1). Patient baseline characteristics are presented in Table 1.

**Table 1:** Patient baseline characteristics in FAS

Characteristics	Patients, *n* (%)
Age	
Median age (range, years)	60 (35–74)
< 50 years	6 (14.0)
50–65 years	20 (46.5)
≥ 65 years	17 (39.5)
Sex	
Female	23 (53.5)
Male	20 (46.5)
Race	
Chinese	43 (100.0)
ECOG PS	
0	6 (14.0)
1	37 (86.0)
*EGFR*-sensitive mutation type	
Exon 19 deletion	28 (65.1)
L858R	13 (30.2)
G719X^a^ and S768I mutations	1 (2.3)
L861Q	1 (2.3)
CNS metastases	
Yes	12 (27.9)
No	31 (72.1)
Prior anti-cancer therapy	
Surgery	1 (2.3)
Chemotherapy	0
Radiotherapy	2 (4.7)
Other ^b^	1 (2.3)

*Abbreviations: CNS* Central nervous system, *ECOG* Eastern Cooperative Oncology Group, *PS* Performance status, *FAS* Full analysis set, *EGFR* Epidermal growth factor receptor

^a^The Cobas® *EGFR* Mutation Test, Version 2, Roche Diagnostics, South Branchburg, NJ, USA, was used for *EGFR* mutations detection, which was not able to further confirm this patient’s specific G719X type

^b^One patient received prior anticancer therapy with traditional Chinese medicine

At the data cutoff date on Dec 23, 2021, all patients had terminated rezivertinib treatment, while 22 (51.2%) patients had progressive disease (PD), 15 (34.9%) patients terminated the study by sponsor due to the data cutoff, 4 (9.3%) patients discontinued due to TEAEs, 1 patient terminated due to investigator decision, and 1 patient withdrew of consent (Fig. 1). The median duration of follow-up was 25.3 (95% CI: 25.0–26.2) months.

### Efficacy

**Fig. 2:**
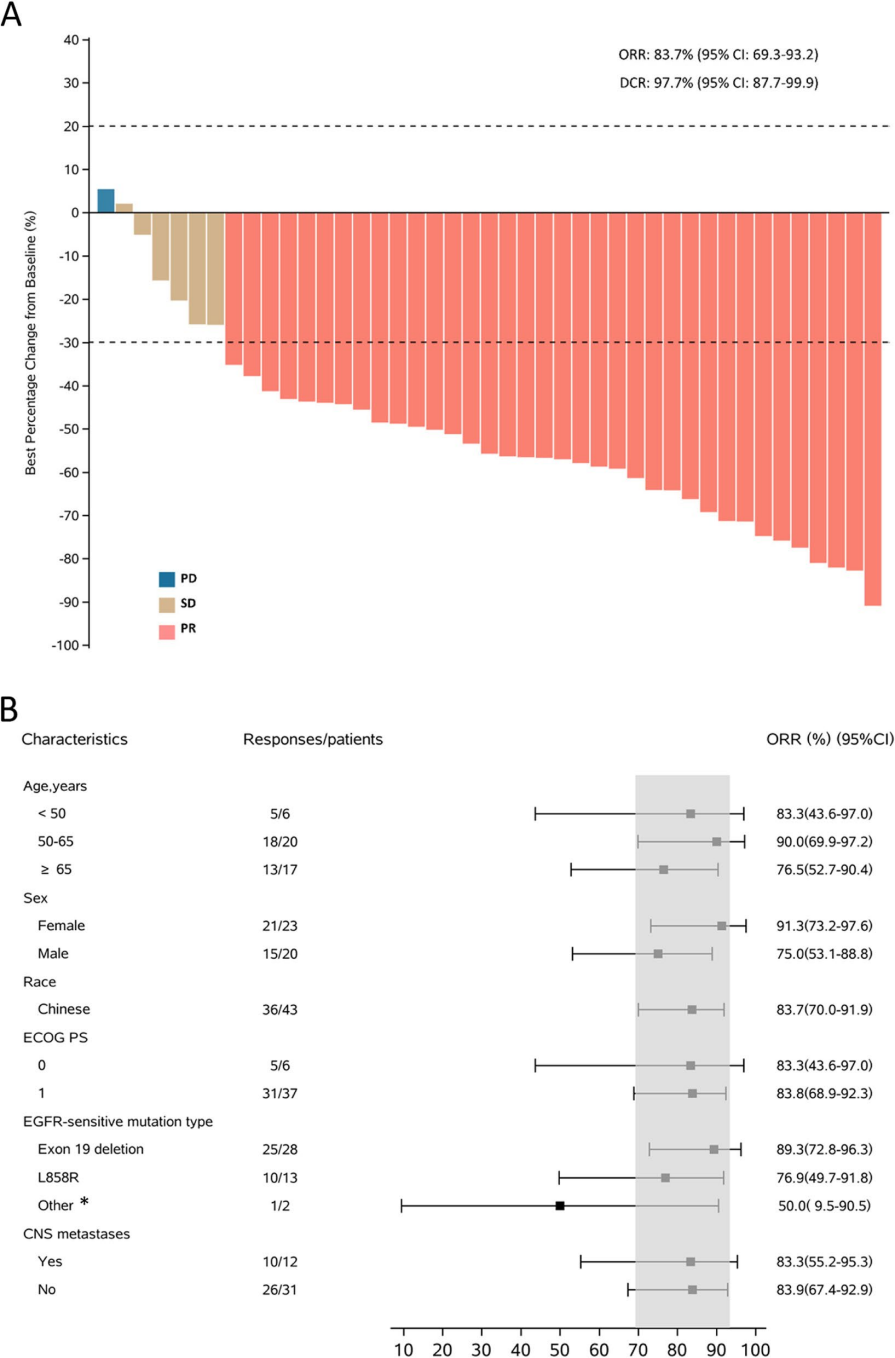
**A** Waterfall plot for BICR-assessed tumor best percentage change from baseline in FAS. The dashed line at 20% represents the boundary for the determination of PD, and the dashed line at − 30% represents the boundary for the determination of PR. Color code: orange for PR; brown for SD; blue for PD. **B** Forest plot for subgroups of patients having BICR-assessed objective responses in FAS. *Other includes one patient with G719X and S768I mutations, and one patient with only L861Q mutation. The Cobas® *EGFR* Mutation Test, Version 2, Roche Diagnostics, South Branchburg, NJ, USA, was used for *EGFR* mutations detection, which was not able to further confirm this patient’s specific G719X type. Abbreviation: FAS, full analysis set; CNS, central nervous system; ECOG, Eastern Cooperative Oncology Group; PS, performance status; BICR, blinded independent central review; ORR, objective response rate; DCR, disease control rate; CI, confidence interval; PR, partial response; SD, stable disease; PD, progressive disease

**Fig. 3:**
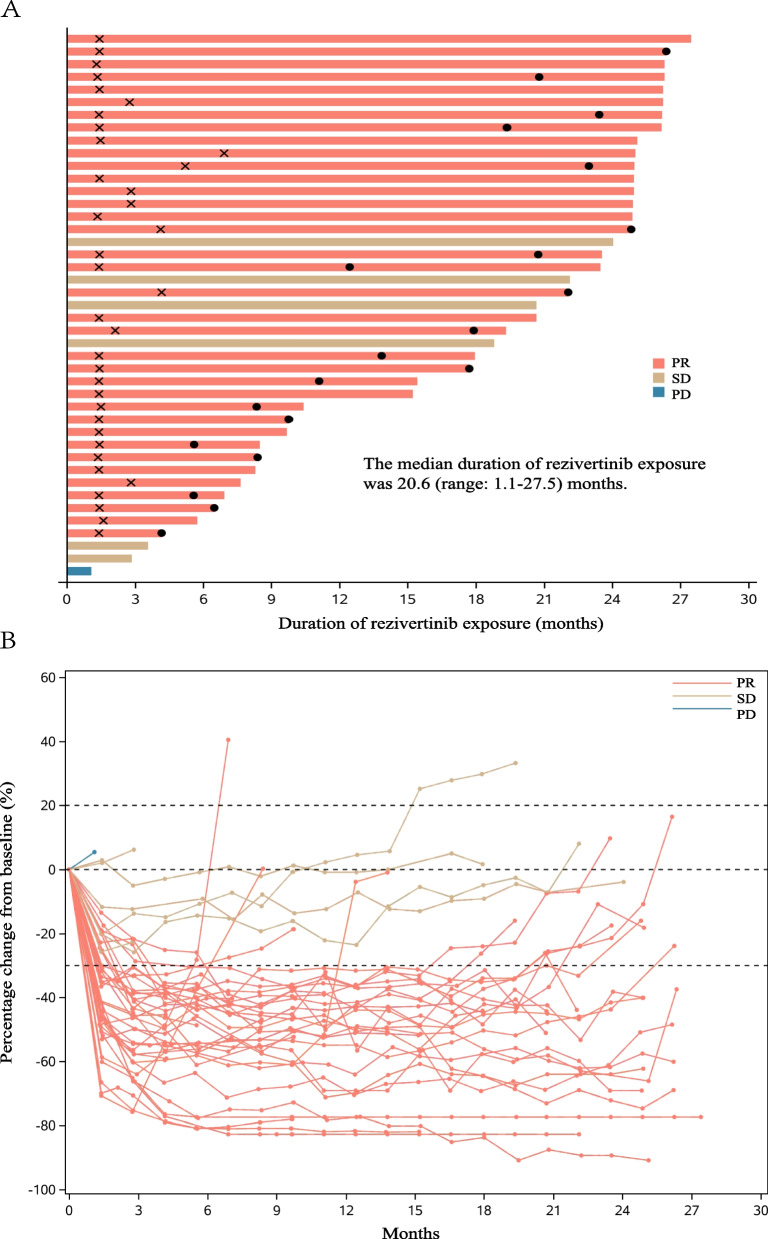
**A** Swimmer plot for the exposure and response duration of rezivertinib in FAS. As per the protocol and the RECIST version 1.1, after the patient’s disease progression, the patient may continue the treatment if investigators considered the patient would still benefit from the study treatment. In patients with a BICR-assessed confirmed objective response, the time when the objective response was first observed is indicated by an × , and the time when the objective response was terminated is indicated by a circle. **B** Spider plot for BICR-assessed percentage change of tumor size from baseline at different time points. Abbreviation: BICR, blinded independent central review; PR, partial response; SD, stable disease; PD, progressive disease; FAS, full analysis set; RECIST: Response Evaluation Criteria in Solid Tumors

**Fig. 4:**
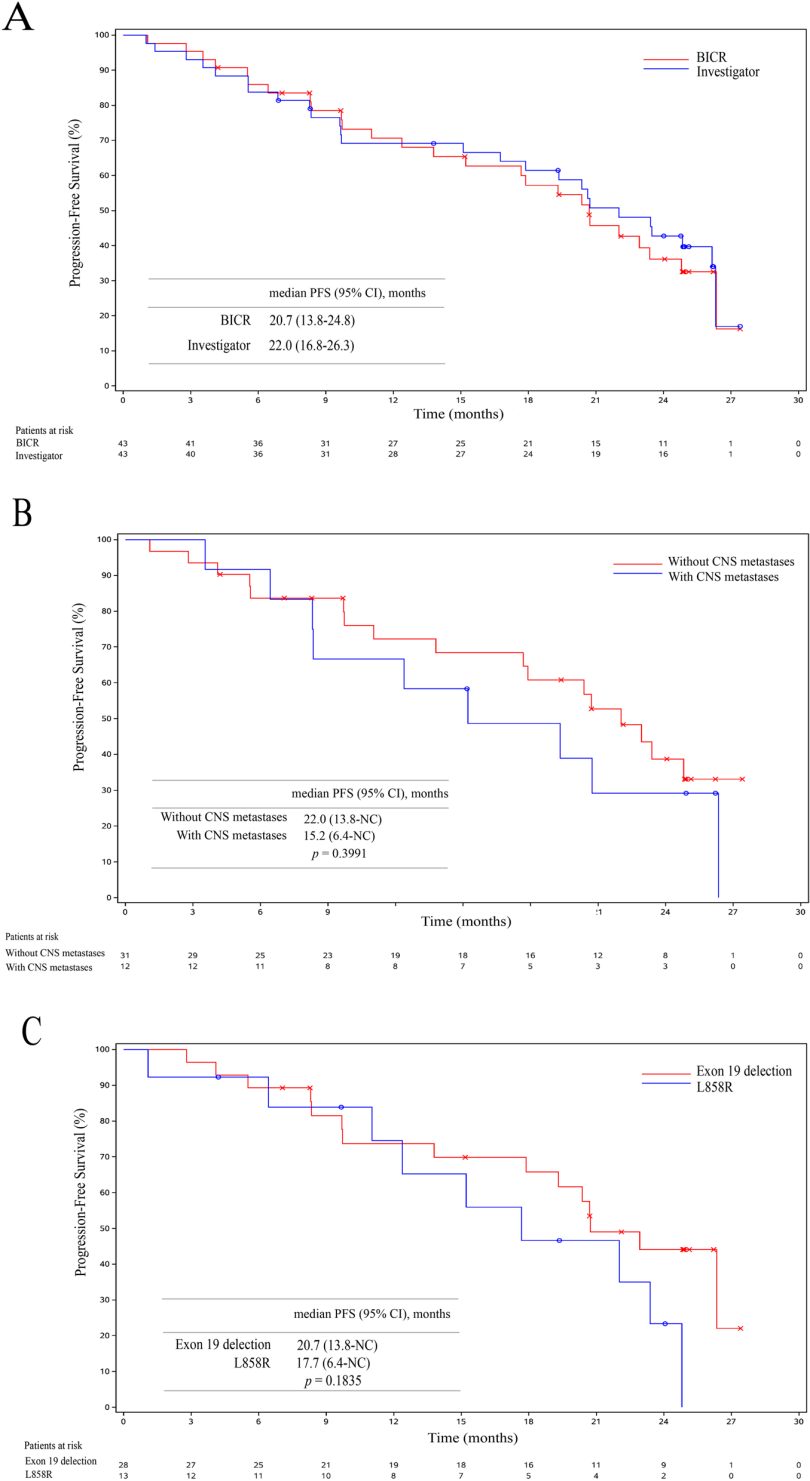
**A** Kaplan–Meier curve for BICR-assessed PFS and investigator-assessed PFS in FAS. **B** Kaplan–Meier curve for BICR-assessed PFS in patients with and without CNS metastases. **C** Kaplan–Meier curve for BICR-assessed PFS in patients with *EGFR* Exon 19 deletion and L858R mutations. Abbreviation: BICR, blinded independent central review; CI, confidence interval; PFS, progression-free survival, FAS, full analysis set; CNS, central nervous system; NC, not calculable

All 43 patients were included in FAS. The BICR-assessed and investigator-assessed ORRs were 83.7% (95% CI: 69.3–93.2%) and 69.8% (95% CI: 53.8–83.0%), respectively. The BICR-assessed and investigator-assessed DCRs were 97.7% (95% CI: 87.7–99.9%) and 95.3% (95% CI: 84.2–99.4%), respectively. The tumor shrinkage was observed in 95.3% (41/43) of patients (Fig. 2A). The forest plot for subgroups of patients having objective responses assessed by BICR in FAS was presented in Fig. 2B. The BICR-assessed and investigator-assessed median DoR were 19.3 (95% CI: 15.8–25.0) months and 19.3 (95% CI: 8.3–25.0) months, respectively. The median duration of rezivertinib exposure was 20.6 (range: 1.1–27.5) months (Fig. 3A), and the BICR-assessed percentage change of tumor size from baseline at different time points was presented in Fig. 3B. At the data cutoff date on Dec 23, 2021, 26 (60.5%) of 43 patients were with PFS events, while 22 (51.2%) had developed PD and 4 (9.3%) patients died before PD. The BICR-assessed and investigator-assessed median PFS were 20.7 (95% CI: 13.8–24.8) months and 22.0 (95% CI: 16.8–26.3) months, respectively (Fig. 4A). Among the 43 patients, 2 patients harbored uncommon *EGFR* mutations. For these 2 patients, one patient was with G719X and S768I mutations, and the BICR-assessed PFS was 5.6 months with the best objective response (BOR) of PR (the Cobas® *EGFR* Mutation Test, Version 2, Roche Diagnostics, South Branchburg, NJ, USA, was used for *EGFR* mutations detection, which was not able to further confirm this patient’s specific G719X type); the other patient was with only L861Q mutation, and the BICR-assessed PFS was 3.6 months with the BOR of SD. Additionally, there were 2 patients accompanied with T790M mutation mistakenly enrolled in this study, one patient was a 67-year-old male with T790M positive and exon 19 deletion mutation who had SD and a PFS of 19.0 months and the other patient was a 65-year-old male with T790M positive and L858R mutation who had PR and a PFS of 21.0 months. At the data cutoff date on Dec 23, 2021, the OS was immature, and 14 (37.2%) patients died, while 27 (62.8%) patients were still alive, one patient was lost to follow-up, and one withdrew of consent. Among 12 (27.9%) of 43 patients with CNS metastases at baseline, the CNS-ORR and CNS-DCR were 50.0% (95%CI: 21.1–78.9%) and 58.3% (95%CI: 27.7–84.8%), respectively. The probability of CNS progression at 12 months was 33.3%. Efficacy results are listed in Table 2. The BICR-assessed median PFS for the patients without and with CNS metastases at baseline were 22.0 (95% CI: 13.8–not calculable [NC]) months and 15.2 (95% CI: 6.4–NC) months, respectively (*p* = 0.3991, Fig. 4B); while the BICR-assessed median PFS for patients with *EGFR* exon 19 deletion mutation and *EGFR* L858R mutation were 20.7 (95% CI: 13.8–NC) and 17.7 (95% CI: 6.4–NC) months, respectively (*p* = 0.1835, Fig. 4C).

**Table 2:** The overall and CNS efficacy of rezivertinib in FAS

Efficacy	BICR-assessed (*n* = 43)	Investigator-assessed (*n* = 43)	Brain Metastases at baseline (*n* = 12)
Response, *n* (%)			
CR	0	0	2 (16.7)
PR	36 (83.7)	30 (69.8)	4 (33.3)
SD	6 (14.0)	11 (25.6)	1 (8.3)
Non-CR/Non-PD	NA	NA	5 (41.7)
PD	1 (2.3)	2 (4.7)	0
ORR, *n* (%)	36 (83.7)	30 (69.8)	6 (50.0) ^a^
95% CI	69.3 to 93.2	53.8 to 83.0	21.1 to 78.9
DCR, *n* (%)	42 (97.7)	41 (95.3)	7 (58.3) ^b^
95% CI	87.7 - 99.9	84.2 - 99.4	27.7 - 84.8
DoR, months	19.3	19.3	NA
Median (95% CI)	15.8 - 25.0	8.3 - 25.0	NA
PFS, months	20.7	22.0	NA
Median (95% CI)	13.8 - 24.8	16.8 - 26.3	NA

*Abbreviations: ORR* Objective response rate, *DCR* Disease control rate, *DoR* Duration of response, *PFS* Progression-free survival, *FAS* Full analysis set, *CNS* Central nervous system, *RANO-BM* Response Assessment in Neuro-Oncology Brain Metastases, *CR* Complete response, *PR* Partial response, *SD* Stable disease, *PD* Progressive disease, *NA* Not applicable

^a^CNS-ORR was defined as the proportion of confirmed CR or PR in brain metastatic lesions evaluated by BICR according to the RANO-BM criteria

^b^CNS-DCR was defined as the proportion of confirmed CR, PR, or SD in brain metastatic lesions evaluated by BICR according to the RANO-BM criteria

### Safety

All 43 patients were included in SS. TEAEs occurred in 42 patients (97.7%), while TRAEs occurred in 40 patients (93.0%) (Additional file 1: Table S1). The most common TRAEs were white blood cell (WBC) decreased (19 of 43, 44.2%), PLT decreased (17 of 43, 39.5%), ANC decreased (13 of 43, 30.2%), anemia (11 of 43, 25.6%), ALT increased (8 of 43, 18.6%), lymphocyte count decreased (6 of 43, 14.0%), AST increased (5 of 43, 11.6%), etc. (Table 3). No ILD was reported. Dose interruption occurred in 2 (4.7%) patients due to TRAEs, and no dose reduction or discontinuation due to TRAEs was recorded.

**Table 3:** The common TRAEs of rezivertinib in SS

	Any grade, *n* (%)	Grade ≥ 3, *n* (%)
WBC decreased	19 (44.2)	0
PLT decreased	17 (39.5)	1 (2.3)
ANC decreased	13 (30.2)	0
Anemia	11 (25.6)	1 (2.3)
ALT increased	8 (18.6)	0
Lymphocyte count decreased	6 (14.0)	1 (2.3)
AST increased	5 (11.6)	0
Leukopenia	4 (9.3)	1 (2.3)
Drug eruption	4 (9.3)	0
Rash	3 (7.0)	0
Thrombocytopenia	3 (7.0)	0
Hypertriglyceridemia	3 (7.0)	0

CTCAE version 4.03, MedDRA version 24.1. Data are indicated as *n* (%). Any grade TRAEs of 5% or higher and grade 3–5 TRAEs of 1% or higher are reported

*Abbreviations: TRAEs* Treatment-related adverse events, *SS* Safety set, *WBC* White blood cell, *PLT* Platelet, *ANC* Absolute neutrophil count, *ALT* Alanine aminotransferase, *AST* Aspartate aminotransferase, *CTCAE* Common Terminology Criteria for Adverse Events, *MedDRA* Medical Dictionary for Regulatory Activities

## Discussion

In this phase IIa study, rezivertinib showed promising efficacy and a manageable safety profile in the first-line treatment of locally advanced or metastatic/recurrent NSCLC patients with *EGFR* mutation, including those with CNS metastases.

There are three third-generation EGFR TKIs available in the People’s Republic of China for advanced NSCLC. Osimertinib, the first third-generation irreversible EGFR TKI, significantly improved the PFS and OS versus first-generation EGFR TKIs gefitinib or erlotinib in patients with *EGFR* mutation-positive NSCLC and had been sequentially approved by both FDA on April 18, 2018, and NMPA on August 31, 2019, as the first-line treatment [21–23]. Another two China innovative third-generation EGFR TKIs, almonertinib and furmonertinib, were approved for the first-line treatment by NMPA on Dec 16, 2021, and Jun 28, 2022, respectively. In the AENEAS study, almonertinib significantly prolonged median PFS (19.3 vs 9.9 months; hazard ratio [HR]: 0.46; *p* < 0.0001) and median DoR (18.1 vs 8.3 months; HR: 0.38; *p* < 0.0001) over gefitinib, with immature OS and acceptable safety [24]. In the furmonertinib FURLONG study, the median PFS (21.0 vs 11.1 months; HR: 0.44; *p* < 0.0001) and median DoR (19.7 vs 10.5 months; HR: 0.39; *p* < 0.0001) were significantly prolonged while compared with gefitinib. The OS data was not yet mature, and the safety was acceptable [25].

The third-generation EGFR TKIs revealed the optimal subgroup efficacy based on patients’ *EGFR* mutation types. In the FLAURA study, the median PFS was 21.4 (95% CI: 16.5–24.3) months and 11.0 (95% CI: 9.7–12.6) months for patients with *EGFR* exon 19 deletion mutation in the osimertinib and first-generation EGFR TKI gefitinib/erlotinib groups, respectively (HR: 0.43 [95% CI: 0.32–0.56]; *p* < 0.001); 14.4 (95% CI: 11.1–18.9) months and 9.5 (95% CI: 8.1–11.0) months for patients with *EGFR* L858R mutation in the two groups, respectively (HR: 0.51 [95% CI: 0.36–0.71]; *p* < 0.001) [21]. In the AENEAS study, among patients with *EGFR* exon 19 deletion mutation, the median PFS for the almonertinib and gefitinib groups was 20.8 (95% CI: 18.1–20.9) months and 12.3 (95% CI: 9.6–13.8) months, respectively (HR: 0.39; *p* < 0.0001), while among patients with L858R mutation, the median PFS were 13.4 (95% CI: 7.3–18.0) months and 8.3 (95% CI: 6.8–9.9) months for the two groups, respectively (HR: 0.60; *p* = 0.0102) [24]. In the FURLONG study, furmonertinib significantly reduced the risk of progression or death while compared with gefitinib with HRs of 0.35 (95% CI: 0.23–0.53; *p* < 0.0001) and 0.54 (95% CI: 0.37–0.77; *p* = 0.0006) among patient with *EGFR* exon 19 deletion and L858R mutations, respectively [25]. Despite being a single-arm study, the rezivertinib phase IIa study was revealed to be associated with long median PFS for patients with *EGFR* exon 19 deletion and L858R mutations (20.7 [95% CI: 13.8–NC] months and 17.7 [95% CI: 6.4–NC] months; *p* = 0.1835), which were consistent with the overall efficacy. Apart from clinical trial results, real-world data of osimertinib from the FLOWER and the ASTRIS global study had demonstrated similar efficacy and safety consistent with its previous clinical studies [26, 27]. More real-world data of other third-generation EGFR TKIs are awaited.

Compared with the first- or second-generation EGFR TKIs, the third-generation EGFR TKIs have improved CNS efficacy. In the FLAURA study, osimertinib reduced the risk of CNS progression or death while compared with the first-generation EGFR TKI gefitinib/erlotinib with an HR of 0.48 (95% CI: 0.26–0.86; *p* = 0.014) in the CNS full analysis set (cFAS) [28]. In the AENEAS study, almonertinib achieved longer median CNS-PFS over gefitinib in cFAS [29.0 vs 8.3 months; HR: 0.323 (95% CI: 0.181–0.576); *p* < 0.0001] [29]. Furmonertinib achieved a median CNS-PFS of 11.6 (95% CI: 8.3–13.8) months for *EGFR* T790M mutated patients in its phase IIb study for the cFAS and further prolonged the median CNS-PFS while compared with gefitinib in cFAS (20.8 vs 9.8 months; HR: 0.40 [95% CI: 0.23–0.71]; *p* = 0.0011) in the FURLONG study [15, 30].

Rezivertinib is one of the novel third-generation EGFR TKIs; the phase IIa study results were consistent with the previous phase I study [17] and further verified by the results of the phase IIb study (*n* = 226) which investigated the efficacy and safety of rezivertinib in patients with locally advanced or metastatic/recurrent *EGFR* T790M mutated NSCLC, including treatment-naïve patients and EGFR TKI previously treated patients [18]. The results of the phase IIb study showed that the ORR was 64.6% (95% CI: 58.0–70.8%), and the median PFS was 12.2 (95% CI: 9.6–13.9) months by BICR. The subgroup efficacy was consistent with the overall efficacy, with the ORR of 70% (95% CI: 61.0–78.0%) and median PFS of 13.9 (95% CI: 11.3–17.9) months for the tissue sample T790M positive group. The median OS was 23.9 (95% CI: 20.0–NC) months in FAS. Furthermore, the median CNS-PFS was 16.6 (95% CI: 11.1–NC) months for the 91 (40.3%) patients with CNS metastases at baseline. The efficacy of this phase IIa study was consistent with previous reported results.

The safety profile of rezivertinib was favorable without new safety signals. Here we would like to discuss three aspects of safety profile. Firstly, dermatological and gastrointestinal toxicities were common for the first-/second-generation EGFR TKIs and osimertinib in the global FLAURA study [21, 31], while rezivertinib mainly presented hematological toxicity which was partially similar to osimertinib among Chinese patients [23]. The top five most common TEAEs were WBC decreased (41%), anemia (38%), rash or acne (37%), PLT decreased (28%), and diarrhea (24%) in the FLAURA China study [23], while that in this phase IIa study of rezivertinib were WBC decreased (44.2%), PLT decreased (41.9%), ANC decreased (32.6%), anemia (30.2%), and ALT increased (20.9%). However, the mechanism of hematological toxicity has not been elucidated. Fortunately, the hematological toxicity was all well-tolerated with a limited effect on the study dose adjustment. Secondly, ALT elevated related to rezivertinib was observed in 8 (18.6%) patients and none of them was grade ≥ 3, while AST elevated related to rezivertinib was observed in 5 (11.6%) patients and none of them was grade ≥ 3. Also, there was no sign indicating that ALT or AST elevated was related to viral hepatitis since two patients with hepatitis B disease at baseline experienced no ALT or AST elevation by the data cutoff date. Thirdly, drug-induced ILD has been considered as an infrequent but non-negligible serious fatal TRAEs that occurred in all generations of EGFR TKIs, and the molecular mechanisms have not been clarified [32]. Among the clinical studies of third-generation EGFR TKIs in the first-line setting, 6 patients with ILD were recorded as osimertinib-related in the global FLAURA study while grade 3 ILD occurred in one patient as severe TRAE in the FLAURA China study [21–23]. For almonertinib and furmonertinib, the ILD was also observed in two and one patient in the AENEAS and the FURLONG studies, respectively [24, 25]. However, in this phase IIa study, no ILD was observed after the median follow-up duration of 23.3 (95% CI: 22.8–23.9) months. Furthermore, throughout the phase I and phase IIb studies of rezivertinib, no ILD has been observed either [17, 18].

There are limitations to this phase IIa study. Firstly, the sample size was limited to only 43 patients and a strict randomized trial with a larger sample size is needed to further confirm the potential efficacy of rezivertinib. Encouragingly, a phase III REZOR study comparing rezivertinib with gefitinib in the first-line setting is ongoing, and the patient enrollment has been completed (NCT03866499). Secondly, there might be a potential bias while comparing with other ethnic patients since this phase IIa study was conducted among Chinese patients only.

## Conclusions

In conclusion, in this study, rezivertinib (BPI-7711) showed promising efficacy and a favorable safety profile for the treatment among the locally advanced or metastatic/recurrent NSCLC patients with *EGFR* mutation in the first-line setting.

## Supplementary Information

**Additional file 1: Table S1. **Safety Summary of rezivertinib in SS.

## Data Availability

The data and materials that support the findings of this study are available from the corresponding author upon reasonable request.

## Abbreviations

AE: Adverse event
ALT: Alanine aminotransferase
ANC: Absolute neutrophil count
APTT: Activated partial thromboplastin time
AST: Aspartate aminotransferase
BICR: Blinded independent central review
BOR: Best objective response
cFAS: CNS full analysis set
CI: Confidence interval
CNS: Central nervous system
CR: Complete response
CTCAE: National Cancer Institute Common Terminology Criteria for Adverse Events
DCR: Disease control rate
DoR: Duration of response
ECG: Electrocardiogram
ECOG: Eastern Cooperative Oncology Group
EGFR: Epidermal growth factor receptor
FAS: Full analysis set
FDA: Food and Drug Administration
HR: Hazard ratio
ILD: Interstitial lung disease
INR: International normalized ratio
NC: Not calculable
NMPA: National Medical Products Administration
NSCLC: Non-small cell lung cancer
ORR: Objective response rate
OS: Overall survival
PD: Progressive disease
PFS: Progression-free survival
PLT: Platelet
PR: Partial response
PS: Performance status
QTcF: QT interval corrected for heart rate using Fridericia's formula
RANO-BM: Response Assessment in Neuro-Oncology Brain Metastases
RECIST: Response Evaluation Criteria in Solid Tumors
RP2D: Recommended phase II dose
SD: Stable disease
SS: Safety set
TEAE: Treatment-emergent adverse event
TKI: Tyrosine kinase inhibitor
TRAE: Treatment-related adverse event
ULN: Upper limit of normal
WBC: White blood cell
