# Supplementary material for: Results of the phase IIa study to evaluate the efficacy and safety of rezivertinib (BPI-7711) for the first-line treatment of locally advanced or metastatic/recurrent NSCLC patients with EGFR mutation from a phase I/IIa study
Source: BMC Med. 2023 Jan 8;21:11. doi: 10.1186/s12916-022-02692-8 (PMC9827694; doi:10.1186/s12916-022-02692-8)
Supplement: Supplementary file 1 — Additional file 1: Table S1. Safety Summary of rezivertinib in SS. [file 12916_2022_2692_MOESM1_ESM.docx]

**Additional File 1**

Table S1. Safety Summary of rezivertinib in SS

| AEs | Patients (n=43) (%) |
| --- | --- |
| TEAEs | 42 (97.7) |
| Grade ≥3 TEAEs | 16 (37.2) |
| TRAEs | 40 (93.0) |
| Grade ≥3 TRAEs | 4 (9.3) |
| Dose interruption due to TEAEs | 3 (7.0) |
| Dose reduction due to TEAEs | 0 |
| Discontinuation due to TEAEs | 3 (7.0) |
| Dose interruption due to TRAEs | 2 (4.7) |
| Dose reduction due to TRAEs | 0 |
| Discontinuation due to TRAEs | 0 |
| Any SAEs | 12 (27.9) |
| Treatment-related SAEs * | 0 |

Note: Data are n (%). * As assessed by the investigators. Abbreviation: AEs, adverse events; TEAEs, treatment-emergent adverse events; TRAEs, treatment-related adverse events; SS, safety set; SAEs, serious adverse events.
